# Robert S. Schwartz, MD, a transformative figure in immunosuppression that revolutionized transplantation

**DOI:** 10.3389/frtra.2023.1258950

**Published:** 2023-08-25

**Authors:** Xian C. Li

**Affiliations:** Immunobiology and Transplant Science Center and Department of Surgery, Houston Methodist Hospital, Houston, TX, United States

**Keywords:** transplantation, immunosuppression, graft rejection, immune tolerance, graft survival

## Abstract

While the transplant community celebrated more than a million transplant patients in the United States, we are reminded that our journey to such a celebratory success is the results of remarkable breakthroughs and brilliant innovators. Among those, immunosuppression drugs are undoubtedly a cornerstone of transplant success, an area where Dr. Robert Schwartz is undeniably a transformative figure and a pioneer. His seminal studies on 6-mercaptopurine in 1959 gave birth to an entirely new specialty of immunosuppression that dramatically accelerated the advancement of clinical organ transplantation.

In 2022, the transplant community celebrated a milestone, i.e., over a million patients transplanted in the United States (1). In hindsight, our journey to such a celebratory success has been extraordinary, a journey that is marked with remarkable breakthroughs and brilliant innovators. Among those, immunosuppression drugs are undoubtedly a cornerstone of transplant success, an area where Dr. Schwartz is undeniably a transformative figure and a pioneer (Figure 1). His seminal studies on 6-mercaptopurine (an analogue of Azathioprine) in 1959 gave birth to an entirely new specialty of immunosuppression, and at a time when graft rejection was universal, his discoveries dramatically accelerated the advancement of clinical organ transplantation (2). As a physician-scientist, Dr. Schwartz had an amazing repertoire of accomplishments: a long-time professor of medicine at Tufts University, chief of hematology at Tufts for nearly 30 years, deputy editor of New England Journal of Medicine for 15 years, recipient of the Peter Medawar prize, Thomas Starzl award, and Stratton award, just to list a few.

**Figure 1:**
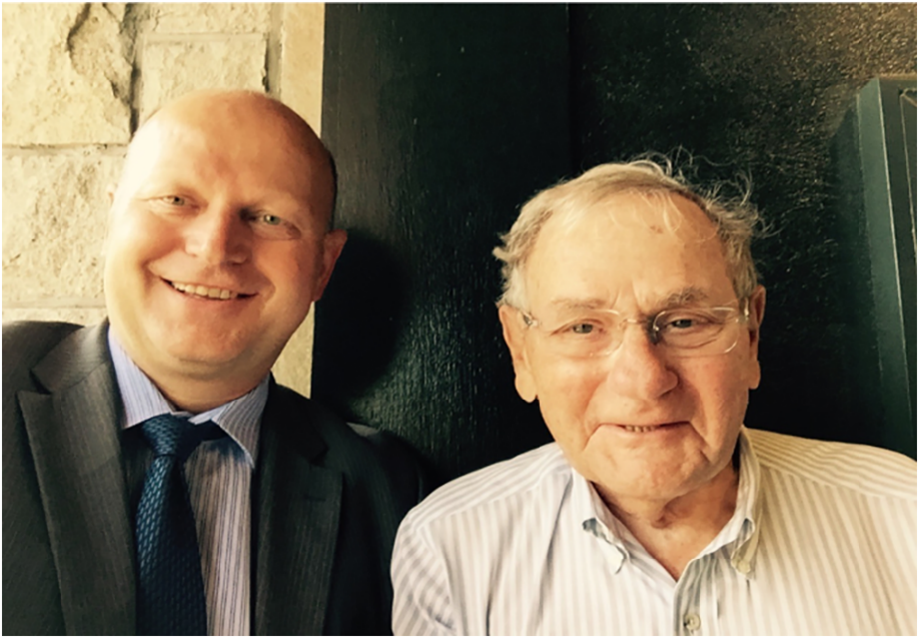
Dr. Robert Schwartz (right), a renowned hematologist and an innovator in transplant immunosuppression. Pictured here with Dr. Stefan Tullius (left) in 2015. (with permission from Dr. Tullius at Brigham and Women's Hospital).

My first encounter with Dr. Schwartz was, by all count, ordinary. In 2000, the international transplant congress (TTS2000) was held in Rome, Italy, where Dr. Schwartz was honored with the Peter Medawar prize, the highest award in the transplant community. I was a freshly minted assistant professor at Harvard with a strong interest in transplant tolerance (3), and like other junior members in the community, I descended to Rome with tremendous hope and excitements. I caught a glimpse of Dr. Schwartz during the award ceremony, but his name was simply ingrained in my mind because of immerse admiration. Interestingly, the opportunity that got me to know him personally was nothing short of extraordinary. In the summer of 2001, we sent our 7-year-old daughter Stephanie to a summer camp in Wellesley, Massachusetts, where she quickly befriended another camper, a lovely girl called Annie McDonald Schwartz. Annie was overjoyed and believed that Stephanie was also adopted from China (both were the only Asians in the camp). Annie demanded that her parents Adam and Michele meet us and arrange playdates, and because of that, both families also became close friends. We then commuted frequently between Newton (where we lived) and Wellesley (where they lived) visiting each other, and little did I know that Adam is the elder son of Dr. Schwartz. At that time, Adam taught creative writing class at Wellesley College and Michele was a professional photographer for the Boston Globe newspaper.

Adam introduced me to his father over a family party one Saturday afternoon in his house at Wellesley. Adam thoughtfully ordered thin crust pizza from Figs Beacon Hill, an upscale restaurant in Boston, saying that thin crust is his father's favorite. That was the occasion that we met and had our first conversion that felt nothing like thin crust. Similar to many conversations that followed, that first one still feels so fresh, enlightening, and profound. I acted like many other junior faculties, and when face to face with a giant in the field, my first goal was to extract as many insights as possible over how to establish a successful career and how to do impactful research. That was also the time that I got a closer peek at his unparalleled style and personality.

It was a pure joy to listen to Dr. Schwartz telling stories and explaining complex concepts in captivating words. When his wife Ruth (in a real estate business) asked why there are T cells and B cells, but no A cells, in immunology, he explained vividly the Thymus and Bursa of Fabricius in chicken, structures where those cells are derived, emphasizing that it took a brilliant English educator Ivan Roitt to coin the words T cells and B cells. I remember the moment that all kids stopped playing and joined the audience, and all cheered with blissful smiles. He had a unique way of commanding words, and his voice certainly had an attention-grabbing tune. I learned later that Dr. Schwartz was repeatedly recognized as the best in his teaching from medical students at Tufts University Medical School, winning 13 annual outstanding teaching awards. He served as deputy editor for the New England Journal of Medicine for almost 15 years, a leading medical journal in the world, attesting to his incredible knowledge and language skills. I read again and again his 1959 Nature paper describing how 6-mercaptopurine as an immunosuppressant that rendered the mature immune system unresponsive to protein antigens (4), the style, accuracy, and precision in the paper are truly exemplary even to this day.

Despite his stature, Dr. Schwartz was remarkably humble and approachable, a trait that is also possessed by many other trailblazers in the field. He often attributed his discovery as simply having been “lucky”. He proclaimed that he is a hematologist, not a transplantor, and less concerned about organ rejection. He insisted that his recognition in the transplant world was just “something nice” to see. Based on his recollection (5), his chief, Dr. Dameshek, assigned him the task of finding new therapies other than total body irradiation to prevent bone marrow allograft rejection. He turned to chemicals known to have activities in lymphoblastic leukemia and wrote to multiple companies requesting materials for his experiments. It turned out that only one company replied and sent him a chemical compound called 6-mercaptopurine. He later learned that 6-mercaptopurine works the best in rabbits, an animal model that he used at that time for all his studies. That was perhaps the reason why he factored “luck” into his landmark findings. The truth of the matter is that all his studies are down to details, well designed, well thought of and accurately presented, leaving practically nothing to “alternative explanations”. He initially found that 6-mercaptopurine rendered a mature immune system “tolerant” to protein antigens and also markedly extended skin graft survival in rabbits (4, 6). Those observations in fact set off an intense chain of reactions that eventually made chemical immunosuppression a clinical reality in relatively a short period of time. An analogue of 6-mercaptopurine Azathioprine was designed thereafter, tested in dogs by Sir Roy Calne and others, and quickly entered the clinic. Some kidney transplant patients, though a small percentage, survived for years when treated with Azathioprine, an outcome never seen before (Figure 2) (7). The enthusiasm generated led to the development of other more powerful immunosuppressive drugs in the 1970s and 1980s that clearly transformed clinical transplantation. The introduction of cyclosporine A and later FK506 resulted in an explosion in the volume as well as the type of organs transplanted (8). Dr. Joseph Murray remarked in his 1990 Nobel laureate that “real breakthrough (in transplantation) came with the introduction of immunosuppression drugs by Schwartz and Dameshek in 1959”.

**Figure 2:**
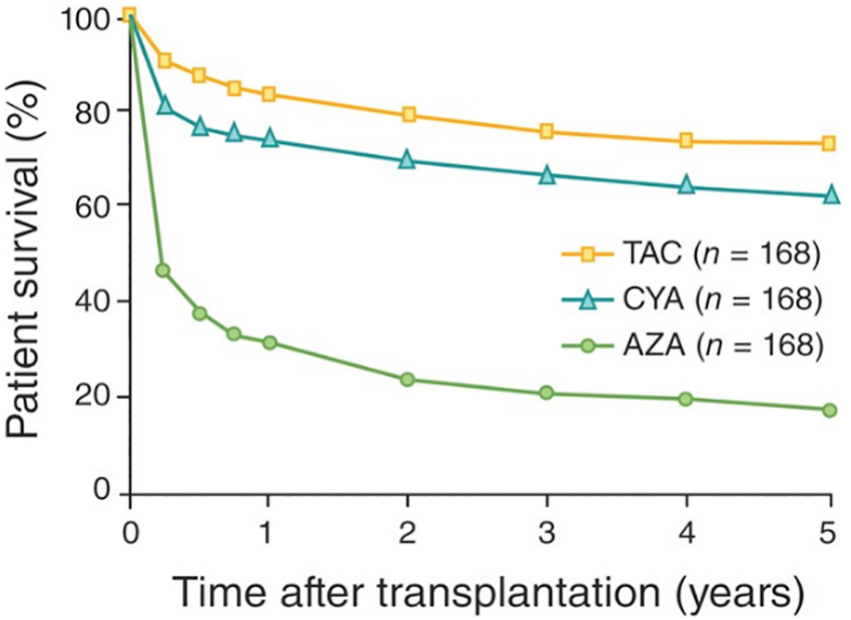
Kidney transplant survival in the clinic in the era of chemical immunosuppression. AZA, azathioprine; CYA, cyclosporine A; TAC, tacrolimus or FK506 (6).

Dr. Schwartz embodied an era in which the United States' biomedical enterprise produced brilliant physician-scientists, an era that we may not see again. When I asked for career advice in our first conversation, he unexpectedly encouraged me to seriously think about going back to the clinic, he correctly predicted the corporatization of medicine and the constrains of fundamental discovery research for future generations. Now more than 20 years later, I am still in awe of his insights. Indeed, we are facing mounting challenges now not only in discovery research, but also the dwindling pool of physician-scientists, who are often bogged down by limited resources, time, and bureaucratic burdens (9).

After his retirement, Dr. Schwartz enjoyed photography, a hobby that he developed from his profession as a hematologist. He often said that “pictures of blood as seeing through microscope are nothing short of astonishing, with different shapes, sizes, and colors”. Thus, it's a natural transition for him in seeing beauty through the lens of a camera. His love of photography also became a shared family affair with his daughter in law Michele, who is a professional photographer. Dr. Schwartz joked that he first met Michele when she was assigned by the Boston Globe to photograph him in the editorial office of New England Journal of Medicine, which is located in the Countway library at Harvard Medical School. Michele also gracefully shared her expertise and equipment with me including high-end camera lenses, and some of them are still with me to this day.

Dr. Schwartz passed away in August 2017 at the age of 89, he will always be remembered as a transformative figure in immunosuppression, a cornerstone of transplant success that we all celebrate today (10).

## Data Availability

The original contributions presented in the study are included in the article/Supplementary Material, further inquiries can be directed to the corresponding author.
